# Magnetic Induction Assisted Heating Technique in Hydrothermal Zeolite Synthesis

**DOI:** 10.3390/ma15020689

**Published:** 2022-01-17

**Authors:** Supak Tontisirin, Chantaraporn Phalakornkule, Worawat Sa-ngawong, Supachai Sirisawat

**Affiliations:** 1 Department of Chemical Engineering, Faculty of Engineering, King Mongkut’s University of Technology North Bangkok, Bangkok 10800, Thailand; chantaraporn.p@eng.kmutnb.ac.th (C.P.); worawat.sa@outlook.com (W.S.-n.); supachai.sirisawat@gmail.com (S.S.); 2 Center of Eco-Materials and Cleaner Technology, King Mongkut’s University of Technology North Bangkok, Bangkok 10800, Thailand

**Keywords:** hydrothermal synthesis, zeolite synthesis, magnetic induction, zeolite X, NaX, scaleup

## Abstract

The magnetic induction assisted technique is an alternative heating method for hydrothermal zeolite synthesis with a higher heat-transfer rate than that of the conventional convection oil bath technique. The research demonstrates, for the first time, the application of the magnetic induction heating technique with direct surface contact for zeolite synthesis. The magnetic induction enables direct contact between the heat source and the reactor, thereby bypassing the resistance of the heating medium layer. A comparative heat-transfer analysis between the two methods shows the higher heat-transfer rate by the magnetic induction heating technique is due to (1) eight-time higher overall heat-transfer coefficient, attributed to the absence of the resistance of the heating medium layer and (2) the higher temperature difference between the heating source and the zeolite gel. Thereby, this heating technique shows promise for application in the large-scale synthesis of zeolites due to its associated efficient heat transfer. Thus, it can provide more flexibility to the synthesis method under the non-stirred condition, which can create possibilities for the successful large-scale synthesis of a broad range of zeolites.

## 1. Introduction

Zeolites are crystalline aluminosilicate microporous materials. They can be natural or synthetic [1]. They have a three-dimensional framework structure, which has an open pore with a size of approximately 0.3–1 nm [2]. Due to their high surface area, numerous active sites, high thermal and chemical stability, and shape selectivity, zeolites are widely used in many industrial applications as adsorbents, ion-exchangers, and catalysts [3,4,5,6,7]. Most of their applications are relevant to environmental remediation and renewable energy for a sustainable society [8,9,10,11,12]. Zeolites are the largest volume used as ion-exchangers for reducing the hardness of the water in detergent application. This revolutionary application prevents the death of living creatures in rivers caused by the traditional phosphate substitute compound, via a process known as “Eutrophication”. Conversely, the highest market value of zeolites is as catalysts, particularly in refineries because of their unique properties.

Due to their numerous benefits and environmental friendliness, zeolites are demanded in high capacity for various applications. However, their production technique still has room to be improved for the high-quality zeolites at a low production cost. Synthetic zeolites can be produced by hydrothermal method at a high temperature and pressure, in an aqueous solution, and in a closed reactor system [13,14,15]. In a small scale, the heat sources are typically provided by a convection hot air oven and an oil bath. Since the heat transport mode is mainly convection, the arising problem is the slow heat transfer across the reactor to the core of the zeolite gel, and there could be heat loss from the heat source to the environment. Magnetic induction heating is a suitable technique for directly heating the zeolite gel system. This technique is applied by oscillating the magnetic field, which causes the electrons in the magnetic material to move around and, consequently, creates a large eddy of electron current [16]. In zeolite production by the hydrothermal method, there are two possible methods for heat generation by magnetic induction: (1) via the conductive properties of the solution [17] and (2) via induction of heat in the magnetic vessel or material which contact with the zeolite gel. This can be used by coating application of the stainless-steel substrate immersed in the zeolite gel. The heat in the substrate is generated by induction of the coil by distance [18]. Alternatively, it can be used by direct contact of the magnetic vessel with the reactor surface. For the first method, the heat is generated in the zeolite solution because the gel solution exhibits conductive property (i.e., the ionic strength) due to the mobile ions. However, the effect of magnetic induction on the formation mechanism and destabilization of the zeolite framework is not clearly known. It might destabilize certain crystalline domains of the zeolite, which are crucial for the success of the zeolite synthesis. The second method is more flexible than the first one. That is, it is not limited only to the conductive liquid. It can be applied to all types of liquids. Applying fast heat transfer to the zeolite gel can reduce temperature gradient in the bulk and can reduce the energy supply for the zeolite production. Importantly, the direct contact of the heat from a magnetic vessel with the reactor system or magnetic material contacting with the zeolite gel will not disturb or destabilize the crystalline domains of the zeolite. Therefore, applying the magnetic induction heating by the direct contact of the magnetic vessel to the zeolite is more practical and potential approach for producing different zeolite types.

This research demonstrates, for the first time, the technique of applying magnetic induction heating by direct surface contact of the magnetic vessel with the reactor surface for the bench-scale zeolite synthesis. NaX type zeolite was hydrothermally synthesized under the non-stirred condition. The temperature profiles and product quality at different points in the reactor were investigated to evaluate the heat-transfer performance. The clarification of their advantages by experimental data and theoretical calculation is essential for elucidating this approach. The heating technique by the conventional convection oil bath was conducted for comparison.

## 2. Materials and Methods

### 2.1. Apparatus Setup and Synthesis of NaX Zeolite

**Figure 1:**
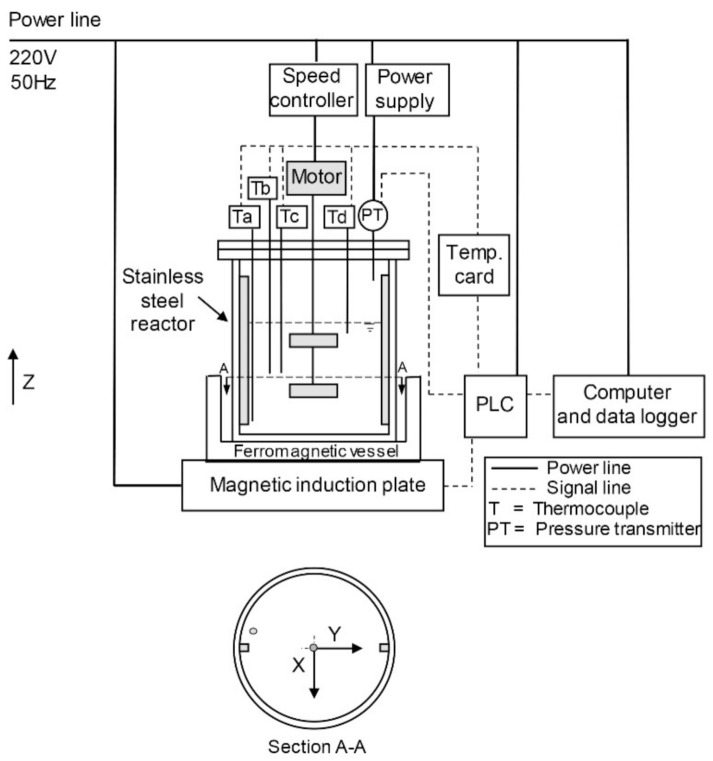
The bench-scale reactor setup with the magnetic induction heating plate.

A 2.5-L turbine-agitator stainless-steel reactor is designed and fabricated. It is placed on the ferromagnetic vessel. The reactor is equipped with a magnetic induction heating plate (max. 1600 W, setting at 600 W), a programmable logic controller (PLC), and a data logger, as shown in Figure 1. The magnetic field is created in this way: the 220 V, 50 Hz alternating current is applied to the induction coil of the magnetic induction heater, creating an alternating electromagnetic field with a frequency of approximately 25 kHz and amplitude in the order of a few micro-tesla (data provided by a commercial supplier). As the alternating magnetic field creates an oscillating electric current and resistance in the ferromagnetic plate, heat is generated inside the metal material by the eddy current. The heat is transferred to the bulk liquid due to the temperature difference between the ferromagnetic plate and the reaction solution. Due to a relatively low magnetic susceptibility of the reaction solution (the CGS volume magnetic susceptibility at 28 °C of −1.752 × 10^−6^), direct induction heating of the solution is expected to be negligible. Four temperature sensors at different locations inside the reactor: depth, radius, and angle positions (Ta = 1.5, Tb = 6.5, Tc = 6.5, Td = 11.5 cm from the base of the reactor; Ta = 6.25, Tb = 6.25, Tc = 3.85, Td = 6.25 cm from the center; Ta = 0, Tb = 30, Tc = 30, Td = 90° from the radius of the circle), are installed to monitor their profiles. A pressure transducer is setup for the detection of the pressure inside the reactor. The apparatus is controlled by the PLC, and data are recorded via the data logger. The NaX zeolite is synthesized with the gel composition 4.42 SiO_2_: 1 Al_2_O_3_: 3.71 Na_2_O: 539 H_2_O. The 7.9 wt.% sodium hydroxide solution (solid NaOH from Merck, 99 wt.%) is added to the sodium aluminate solution (homemade, 10.9 wt.% Al_2_O_3_, 20.5 wt.% NaOH, 68.6 wt.% H_2_O) and stirred for 15 min in the beaker. The 7.9 wt.% sodium hydroxide solution is added to sodium silicate solution (Panreac, 27.4 wt.% SiO_2_, 8.3 wt.% Na_2_O, 64.3 wt.% H_2_O) and stirred for 15 min in the beaker. Then, the two solutions are mixed in the reactor with the total gel of ca. 1.5 L. The gel is stirred with the impeller at 290 rpm for 1 h and subjected to static ageing for 16 h. The reaction occurs at 100 °C for 8 h under the non-stirred or static condition. The temperature and pressure inside the reactor are monitored throughout the synthesis. After the reaction is complete, the solid zeolite product at two different points (the rim and center of the reactor) and the rest (bulk) are separately collected. The zeolite is filtered, washed with deionized water, and dried in the oven at 100 °C for 8 h. The phase and crystallinity of the zeolite are determined by X-ray diffraction spectroscopy (XRD). The chemical composition is analyzed by X-ray fluorescence spectroscopy (XRF). The textural properties are investigated by low-temperature N_2_-adsorption. The analysis of heat transfer using the oil bath technique (2000 W, max. temp. 95 °C) for zeolite synthesis is conducted for comparison with the magnetic induction heating technique. The immersed depth of the reactor in the oil bath is 120 mm.

### 2.2. Characterization of the NaX Zeolite

The magnetic susceptibility of the reaction solution is measured at 28 °C using a magnetic susceptibility balance (Sherwood Scientific, MSB MK1, Cambridge, UK). The obtained zeolite is characterized by powder X-ray diffraction spectroscopy (XRD, Bruker, AXS model D8 Advance, Karlsruhe, Germany) with CuKa (λ = 1.544 A) operating at 40 kV and 30 mA. The measurement runs with 2 theta from 5 to 50 with a step size of 0.02 and a scan speed of 1 s. The specific surface area and pore volume are determined by low-temperature N_2_-adsorption at −196 °C (Microtrac MRB, BELSORP-mini, Osaka, Japan). Before determining the adsorption isotherm, the sample is pretreated at 200 °C for 12 h (BELPREP-flow) to remove the humidity in the zeolite. The specific surface area is calculated by the Brunauer-Emmett-Teller (BET) method in the pressure range of p/p_0_ ≈ 0.03–0.1. The total pore volume is determined by adsorption branch isotherm at p/p_0_ ≈ 0.98. The crystal morphology is revealed using field emission scanning electron microscopy (FESEM, JEOL, JSM-7610, Tokyo, Japan). The elemental composition of the zeolite is measured by X-ray fluorescence spectrometry (XRF, Bruker, S8 TIGER, Karlsruhe, Germany).

## 3. Results and Discussion

### 3.1. The Synthesis of the NaX Zeolite

**Figure 2:**
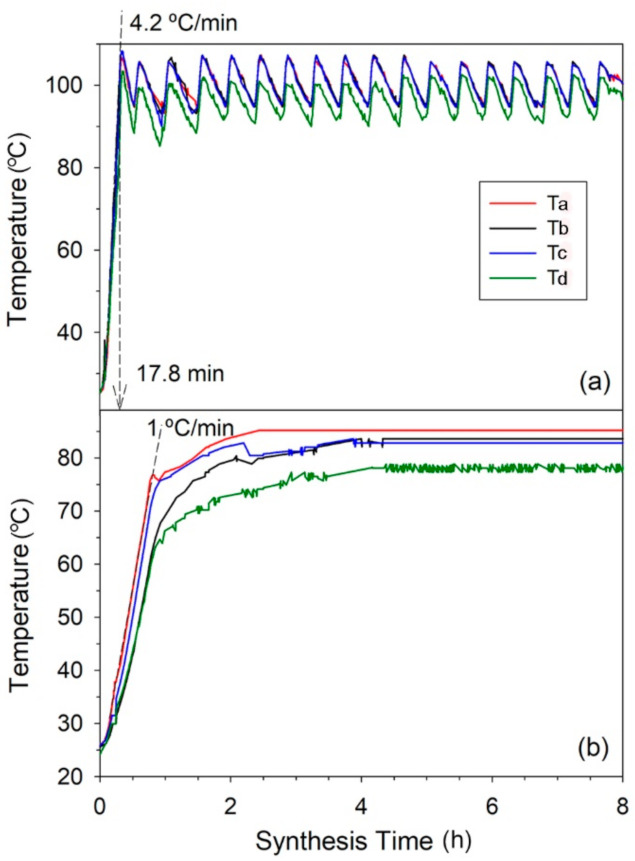
The temperature profiles of the reaction mixture of NaX zeolite for non-stirred synthesis by: (**a**) magnetic induction heating; (**b**) convection oil bath heating.

**Figure 3:**
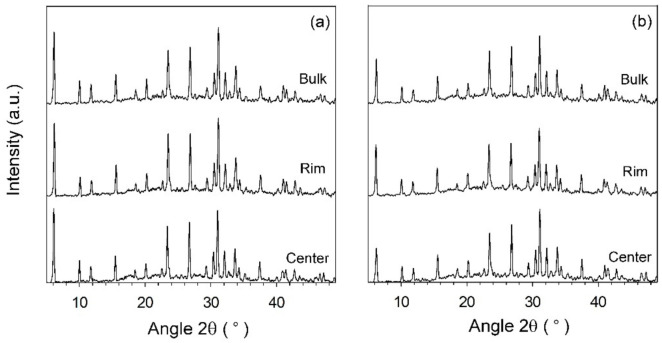
The XRD patterns of NaX zeolite sampled at different locations, synthesized by: (**a**) magnetic induction heating; (**b**) convection oil bath heating.

The temperature profiles of the zeolite gel at different locations in the hydrothermal reactor by magnetic induction heating source and the convection oil bath heating source are shown in Figure 2a,b, respectively. In magnetic induction heating, the ferromagnetic vessel is the heating source, which directly conducts heat to the stainless-steel reactor. Thus, the zeolite mixture is rapidly heated. The four temperatures rapidly rise to the target temperature of 100 °C within 17.8 min at a heating rate of 4.2 °C/min. The temperatures at the different locations of Ta, Tb, and Tc (deep side) show similar profiles at average temperature ca. 100 °C, while Td (shallow side) stays rather at a lower temperature of ca. 96 °C. However, its maximum reaches the target temperature. With oil bath heating, the temperatures slowly rise to the target temperature of 95 °C at a heating rate of 1 °C/min. This is due to the slow heat transfer by natural convection of the oil fluid, which necessitates heating the oil body. All temperatures do not achieve the target temperature (Ta is at the highest of 85 °C). The end temperatures are different. This indicates that the heat loss to the environment easily occurs, resulting in inefficient heat transfer. This shows the magnetic induction heating promotes a high heat-transfer rate and minimizes the heat loss. The zeolite products synthesized by two different heating techniques are sampled at different locations in the reactor: rim, center, and the rest of the bulk. In Figure 3, all samples correspond to the FAU framework of NaX zeolite [19]. Figure 3a shows the XRD patterns of NaX by the magnetic induction heating. The similar patterns and intensities of three samples at different locations in the reactor signify that the mass transfer in the zeolite gel is good. This corresponds to efficient heat transfer by homogenous temperature profiles inside the reactor. Differently, the XRD patterns of samples synthesized by oil bath heating present irregular intensities with the lowest one at the center position of the reactor as shown in Figure 3b. This results by inefficient mass and heat transfer from the heat source to the content in the reactor core.

**Figure 4:**
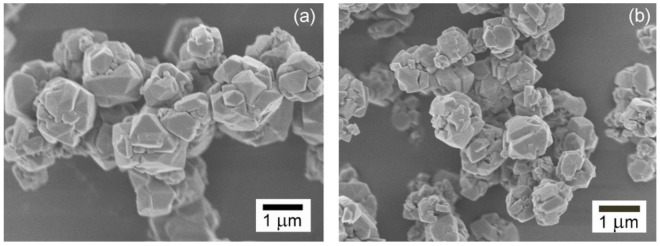
The crystal morphologies of NaX zeolites synthesized by: (**a**) magnetic induction heating; (**b**) convection oil bath heating.

**Figure 5:**
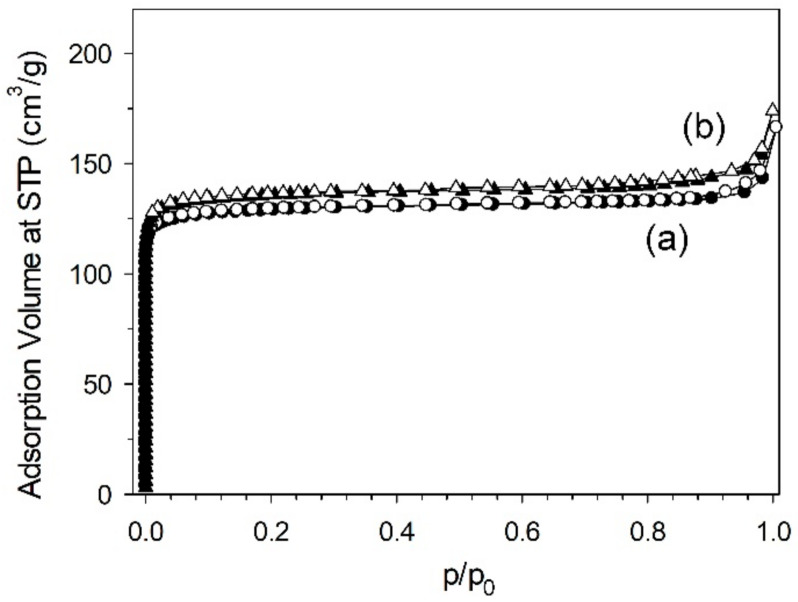
The N_2_ adsorption and desorption isotherms of NaX zeolites synthesized by: (**a**) magnetic induction heating; (**b**) convection oil bath heating. (Closed symbol: adsorption; open symbol: desorption).

The amount of zeolite product obtained by the magnetic induction heating is 51.6 g, with a 56% yield referring to aluminium element, with a molar ratio of Si/Al = 1.14. By the scanning electron microscopy, the bulk NaX zeolite reveals the intergrowth among octahedral and irregular-shape morphologies with crystal size of ca. 1–1.2 μm as shown in Figure 4a. It possesses specific surface area of 495 m^2^/g and pore volume of 0.23 cm^3^/g. Whereas, the zeolite synthesized by the oil bath heating is 56.9 g with a 62% yield referring to aluminium, with a molar ratio of Si/Al = 1.22. The crystal morphology is similar to that of the zeolite synthesized by the magnetic induction heating, but its size is smaller ca. 0.8–1 μm (Figure 4b). This shows slightly lower dispersion of the crystal size. Smaller crystal size can contribute to larger contact surface area. The specific surface area and pore volume are 520 m^2^/g and 0.24 cm^3^/g. The isotherms in both NaX zeolites are presented in Figure 5.

### 3.2. Comparative Study of the Heat-Transfer Efficiency Using Magnetic Induction and Convection Oil Bath Heating Techniques

**Figure 6:**
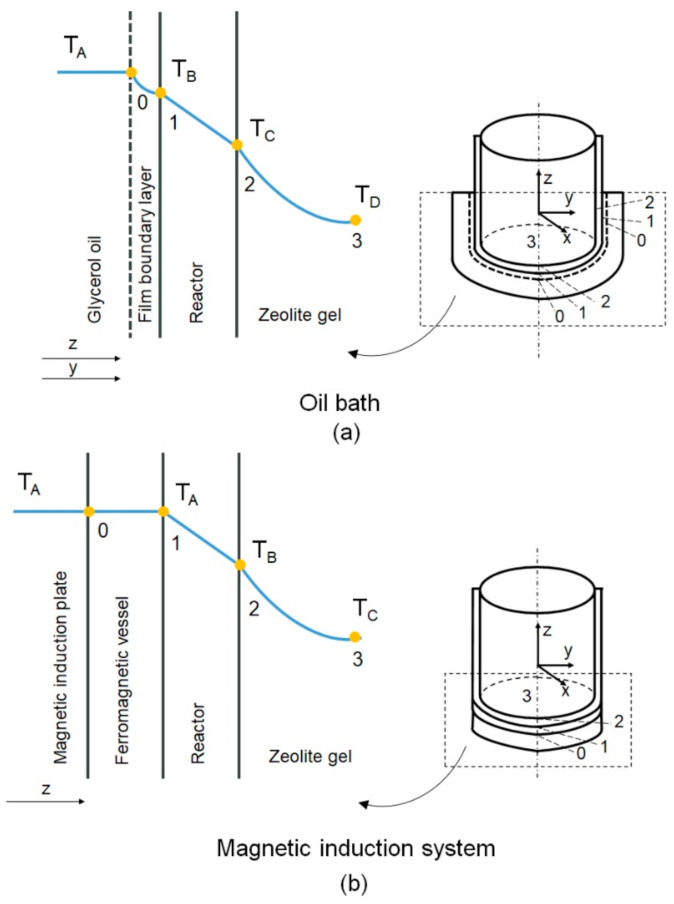
The temperature gradients: (**a**) in the glycerol oil bath heating system; (**b**) in the magnetic induction heating system.

The magnetic induction heating technique is compared with the conventional convection oil bath heating. The geometry of the reactor vessel has a cylindrical shape. However, the cartesian coordinate system is used to describe the space and directions of heat transfer in the reactor vessel because of the symmetry of the properties around the *z*-axis (independent of the angular positions). The representation of the cartesian coordinate system in the cylindrical vessel is shown in Figure 6. This figure illustrates the temperature gradients from the heat sources to the bulk of zeolite gel in the reactor. In the oil bath heating, the heat transfers in the z and y axis. There are at least three resistances to heat transfer (Figure 6a): Region 0–1 where the reactor bottom and wall is in contact with oil ($T_{oil}$), and the heat transfer at the boundary is governed by Newton’s law of cooling; Region 1–2 in which heat is conducted through the stainless steel and the heat transfer is governed by Fourier’s law; Region 2–3 where the reactor bottom and wall is in contact with the zeolite gel at ambient temperature ($T_{zeolite\;gel}$), and the heat transfer at the boundary is governed by Newton’s law of cooling.

The heat-transfer equation for the convection oil bath heating is given in Equation (1):

(1) $$Q_{oil\;bath}\;=\;\frac{∆T_{oil\;bath}U_{oil\;bath}}{\frac{1}{A}}\;=\;\frac{∆T_{oil\;bath}}{\frac{1}{h_{1}A}+\frac{L}{kA}+\frac{1}{h_{2}A}}$$

where

$Q_{oil\;bath}$ is the amount of heat flow in the oil bath heating system (W)

$∆T_{oil\;bath}$ is the temperature difference between $T_{oil}$ and $T_{zeolite\;gel}$, 70 (K)

$U_{oil\;bath}$ is the overall heat-transfer coefficient in the glycerol oil bath heating system (W/m^2^·K)

A is the heat-transfer surface area of the reactor (m^2^)

L is the thickness of the reactor, 0.003 (m)

$h_{1}$ is the free convective heat-transfer coefficient of the glycerol oil (Region 0–1) (W/m^2^⋅K)

$h_{2}$ is the free convective heat-transfer coefficient of the zeolite gel (Region 2–3) (W/m^2^⋅K)

k  is the thermal conductivity of the stainless steel (Region 1–2), 16 (W/m⋅K)

$U_{oil\;bath}$ is calculated according to Equation (2):

(2) $$U_{oil\;bath}\;=\;\frac{1}{\frac{1}{h_{1}}+\frac{L}{k}+\frac{1}{h_{2}}}$$

In convection oil bath heating, $U_{oil\;bath}$ is determined from both horizontal plate and vertical plane/cylinder because the glycerol oil encompasses the reactor bottom and side wall as shown in Figure 6a. For the horizontal plate of the reactor bottom, $h_{1}$ can be calculated according to the relation of dimensionless groups of Nusselt number (Nu) in Equation (3), when the product of the Grashof number (Gr) and the Prandtl number (Pr) is less than 2 × 10^8^ [20].

(3) $$Nu\;=\;0.13{\left(Gr\cdot Pr\right)}^{1/3}$$

(4) $$h_{1}\;=\;\frac{k_{1}}{l}0.13{\left(Gr\cdot Pr\right)}^{1/3}$$

where

$k_{1}$ is the heat conductivity of glycerol oil at reference temperature (W/m⋅K)

l is the characteristic length of heat-transfer surface (m)

The physical properties are determined at the reference temperature according to Equation (5) [21,22,23].

(5) $$T_{e}\;=\;T_{w}-0.25\left(T_{w}-T_{\infty }\right)$$

where

$T_{e}\;$ is the reference temperature (K)

$T_{w}\;$ is the average wall temperature (K)

$T_{\infty }$ is the free-stream temperature (K)

The Grashof number is calculated according to Equation (6) to be 15,602.

(6) $$Gr\;=\;\frac{l^{3}\rho^{2}g\beta \Delta T}{{µ}^{2}}$$

where

l is the characteristic length of heat-transfer surface for horizontal plate, 0.035 (m)

ρ is the density of glycerol oil at reference temperature, 1225 (kg/m^3^)

g is the gravitational acceleration (m/s^2^)

β is the coefficient of thermal expansion of glycerol oil, 5.65 × 10^−4^ (1/K)

∆T is the temperature difference of heated surface and ambient temperature, 70 (K)

μ is the dynamic viscosity of glycerol oil at reference temperature, 0.04 (kg/m·s)

The Prandtl number can be determined according to Equation (7) to be 377.

(7) $$Pr\;=\;\frac{C_{p}\mu }{k_{1}}$$

where

$C_{p}$ is the specific heat capacity of glycerol oil at reference temperature, 2680 (J/kg·K)

μ is the dynamic viscosity of glycerol oil at reference temperature, 0.04 (kg/m·s)

$k_{1}$ is the heat conductivity of glycerol oil at reference temperature, 0.284 (W/m·K)

Therefore, in the horizonal plate, the free convective heat-transfer coefficient of the glycerol oil bath, $h_{1}$, can be calculated to be 190 W/m^2^·K.

The free convective heat-transfer coefficient for the zeolite gel in the non-stirred reactor, $h_{2}$, can be calculated according to Equations (8) and (9), when Gr·Pr is less than 2 × 10^8^ [20].

(8) $$Nu\;=\;0.13{\left(Gr\cdot Pr\right)}^{1/3}$$

(9) $$h_{2}\;=\;\frac{k_{2}}{l}0.13{\left(Gr\cdot Pr\right)}^{1/3}$$

where

$k_{2}$ is the heat conductivity of the zeolite gel at reference temperature (W/m·K)

l is the characteristic length of heat-transfer surface for horizontal plate (m)

The Grashof number and the Prandtl number in this case, can be calculated according to Equations (10) and (11), respectively.

(10) $$Gr\;=\;\frac{l^{3}\rho^{2}g\beta ∆T}{\mu^{2}}$$

(11) $$Pr\;=\;\frac{C_{p}\mu }{k_{2}}$$

where

l is the characteristic length of the heat-transfer surface for horizontal plate, 0.035 (m)

ρ is the density of the zeolite gel at reference temperature, 1167 (kg/m^3^)

g is the gravitational acceleration (m/s^2^)

β is the coefficient of thermal expansion of the zeolite gel, 5.11 × 10^−4^ (1/K)

∆T is the temperature difference of the heated surface and ambient temperature, 70 (K)

μ is the dynamic viscosity of the zeolite gel at reference temperature, 0.0023 (kg/m·s)

$C_{p}$ is the specific heat capacity of the zeolite gel at reference temperature, 4190 (J/kg·K)

$k_{2}$ is the heat conductivity of the zeolite gel at reference temperature, 0.669 (W/m·K)

Thus, Gr and Pr are calculated as 3.87 × 10^6^ and 14.4, respectively. Substituting $k_{2}$, l, Gr, and Pr, $h_{2}$ is calculated to be 949 W/m^2^·K. Therefore, in horizontal plate, the overall heat-transfer coefficient in the glycerol oil bath heating,$\;U_{oil\;bath}$, is calculated to be 154 W/m^2^·K.

The $\;U_{oil\;bath}$ at vertical plane/cylinder (side wall of the reactor) is determined based on Equation (2). The $h_{1}$ is calculated according to the relation of dimensionless group of Nu in Equations (12) and (13) when 10^4^ ≤ Gr·Pr ≤ 10^9^ (Gr·Pr = 2.37 × 10^8^) [20].

(12) $$Nu\;=\;0.59{\left(Gr\cdot Pr\right)}^{1/4}$$

(13) $$h_{1}\;=\;\frac{k_{1}}{l}0.59{\left(Gr\cdot Pr\right)}^{1/4}$$

where

$h_{1}$ is the free convective heat-transfer coefficient of the glycerol oil for vertical plane/cylinder (Region 0–1) (W/m^2^·K)

$k_{1}$ is the heat conductivity of glycerol oil at reference temperature (W/m·K)

l is the characteristic length of heat-transfer surface for vertical plane/cylinder, 0.12 (m)

Thus, the $h_{1}$ is calculated to be 173 W/m^2^·K. The $h_{2}$ is calculated according to the relation of dimensionless group of Nu in Equations (14) and (15) for a vertical plane/cylinder when 10^9^ ≤ Gr·Pr ≤ 10^13^ (Gr·Pr = 2.25 × 10^9^) [20].

(14) $$Nu\;=\;0.021{\left(Gr\cdot Pr\right)}^{2/5}$$

(15) $$h_{2}\;=\;\frac{k_{2}}{l}0.021{\left(Gr\cdot Pr\right)}^{2/5}$$

where

$h_{2}$ is the free convective heat-transfer coefficient of the zeolite for vertical plane/cylinder (Region 0–1) (W/m^2^·K)

$k_{2}$ is the heat conductivity of zeolite at reference temperature (W/m·K)

l is the characteristic length of heat-transfer surface for vertical plane/cylinder, 0.12 (m).

Thus, the $h_{2}$ is calculated to be 645 W/m^2^·K. From Equation (2), the $\;U_{oil\;bath}$ in vertical plane/cylinder is 133 W/m^2^·K. Therefore, $U_{oil\;bath}\;$ of the total system can be determined by the proportion of the contact surface, is 138 W/m^2^·K.

When employing the magnetic induction heating system, there are two resistances to heat transfer as shown in Figure 6b: Region 1–2, in which heat is conducted through the stainless steel, and the heat transfer is governed by Fourier’s law; Region 2–3, where the reactor bottom is in contact with the zeolite gel at ambient temperature ($T_{zeolite\;gel}$), and the heat transfer at the boundary is governed by Newton’s law of cooling. Contrarily, in Region 0–1, the heat is generated by induction, and the reactor bottom is in contact with the bottom of the ferromagnetic vessel at constant temperature ($T_{induction}$). There is no resistance at this region.

The heat-transfer equation for the magnetic induction heating is given in Equation (16):

(16) $$Q_{induction}\;=\;\frac{∆T_{induction}U_{induction}}{\frac{1}{A}}\;=\;\frac{∆T_{induction}}{\frac{L}{kA}+\frac{1}{h_{3}A}}$$

where

$Q_{induction}$ is the amount of heat flow in the magnetic induction heating system (W)

$∆T_{induction}$ is the temperature difference between $T_{induction}$ and $T_{zeolite\;gel}$, 223 (K)

$U_{induction}$ is the overall heat-transfer coefficient in the magnetic induction heating system (W/m^2^·K)

A is the area of the reactor bottom (m^2^)

L is the thickness of the reactor bottom, 0.003 (m)

$h_{3}$ is the free convective heat-transfer coefficient of the zeolite gel for the non-stirred reactor (Region 2–3) (W/m^2^·K)

k is the thermal conductivity of the stainless steel (Region 1–2), 16 (W/m·K)

$U_{induction}$ is calculated according to Equation (17):

(17) $$U_{induction}\;=\;\frac{1}{\frac{L}{k}+\frac{1}{h_{3}}}$$

$h_{3}$ can be calculated according to Equations (18) and (19), when Gr·Pr < 2 × 10^8^.

(18) $$Nu\;=\;0.13{\left(Gr\cdot Pr\right)}^{1/3}$$

(19) $$h_{3}\;=\;\frac{k_{3}}{l}0.13{\left(Gr\cdot Pr\right)}^{1/3}$$

With the heat input of the induction is 600 W, heating time is 3 min, $T_{induction}$ is 521 K, and $T_{zeolite\;gel}$ is 298 K, $∆T_{induction}$ is calculated as 223 K. Substituting Gr, Pr, $k_{3}$, and l, $h_{3}$ is determined as 1396 W/m^2^·K. Thus, the overall heat-transfer coefficient in the magnetic induction heating, $U_{induction}$, is calculated as 1106 W/m^2^·K.

Finally, by substituting all values into Equation (16), the $Q_{induction}$ is calculated to be 3848 W, and $Q_{oil\;bath}$ is 664 W. The calculation shows that $Q_{induction}$ is greater than $Q_{oil\;bath}$ due to two factors: (1) the overall heat transfer coefficient and (2) the temperature difference between the heating source and zeolite gel. Firstly, $U_{induction}$ is eight times greater than $U_{oil\;bath}$ as the magnetic induction system does not depend on the free convective heat transfer of the glycerol oil heating medium. The overall heat-transfer coefficient is in analogy with the electrical conductance in the electrical circuit. The higher the electrical conductance, the higher the electrical current flow. Similarly, the higher the overall heat-transfer coefficient, the higher the heat transfer rate. Secondly, $∆T_{induction}>∆T_{oil\;bath}$ as the magnetic inductor can produce a surface temperature of the ferromagnetic vessel much greater than the oil temperature. The temperature difference in heat transfer is in analogy with the voltage potential in the electrical circuit. The higher the voltage, the higher the electrical current flow. Similarly, the higher the temperature difference, the higher the heat-transfer rate.

In addition, the energy supplies of both heating methods are determined based on one gram of zeolite production as 84.6 kJ for magnetic induction heating and 258 kJ for convection oil bath heating. It shows the advantage in energy saving by applying the induction heating comparing to the convection glycerol oil bath heating. The energy supply is three times lower.

As a result, the high heat-transfer rate produced by the magnetic induction heating could give an opportunity for the non-stirred method (without agitation) in the zeolite synthesis system especially for large-scale production. Typically, the large-scale synthesis needs stirring the zeolite gel, e.g., by impeller, during the reaction to increase the efficiency of the heat transfer in the reaction content. Therefore, the magnetic induction heating technique can create possibility to the successful synthesis of broad ranges of zeolites including the structural types that prefer to form in the static condition.

## 4. Conclusions

The magnetic induction assisted technique is an alternative heating method for the hydrothermal synthesis of zeolites with a high heat-transfer rate and a more uniform temperature distribution. This technique, which involves direct contact between the surface of the heat source and the reactor, exhibits a higher heat-transfer rate than that of the conventional convection oil bath technique. This is due to (1) the eight-time higher overall heat transfer coefficient, attributed to the absence of the resistance of the heating medium layer and (2) the higher temperature difference between the heating source and the zeolite gel. It results more energy saving (three-time lower) by the magnetic induction heating than that by the convection oil bath. The magnetic induction heating by direct surface contact shows promising application in the large-scale zeolite synthesis due to its efficient heat transfer and energy saving. The efficient heat transfer from the heating source to the zeolite gel provides more flexibility to the zeolite synthesis under non-stirred condition. Thereby, it can give possibilities for the successful synthesis of various zeolites in the large-scale production.

## Data Availability

Data sharing is not applicable to this article.
